# Synthesis of *syn*-γ-Amino-β-hydroxyphosphonates by Reduction of β-Ketophosphonates Derived from L-Proline and L-Serine 

**DOI:** 10.3390/molecules15031291

**Published:** 2010-03-04

**Authors:** Mario Ordóñez, Selene Lagunas-Rivera, Emanuel Hernández-Núñez, Victoria Labastida-Galván

**Affiliations:** Centro de Investigaciones Químicas, Universidad Autónoma del Estado de Morelos, Av. Universidad 1001, 62209 Cuernavaca, Mor., México

**Keywords:** β-ketophosphonates, diastereoselective reduction, γ-amino-β-hydroxy-phosphonates

## Abstract

The reduction of γ-*N*-benzylamino-β-ketophosphonates **6** and **10**, readily available from L-proline and L-serine, respectively, can be carried out in high diastereoselectivity with catecholborane (CB) in THF at -78 °C to produce the *syn*-γ-*N*-benzylamino-β-hydroxyphosphonates **11** and **13** as a single detectable diastereoisomer, under non-chelation or Felkin-Anh model control.

## 1. Introduction

**Figure 1:**
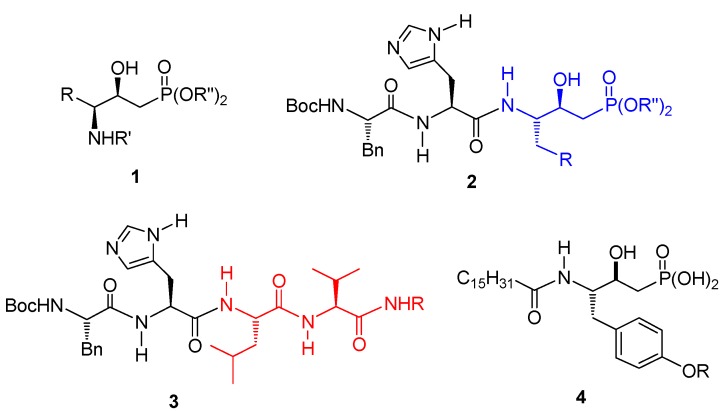
Structures of compounds **1–4**.

Aminoalkylphosphonic acids are structurally analogous to the amino acids, obtained by isosteric substitution of the planar and less bulky carboxylic acid (CO_2_H) group by a tetrahedral phosphonic acid (PO_3_H_2_) functionality. Several aminophosphonic, aminophosphinic and aminophosphonous acids have been isolated from various natural sources, either as free amino acids or as constituents of more complex molecules [1,2,3,4]. In this context, γ-amino-β-hydroxyphosphonates such as **1** (Figure 1) have resulted in unique phosphate mimics with resistance to phosphatase hydrolysis [5,6]. The γ-amino-β-hydroxyphosphonates **1** have been also used in the synthesis of complex molecules **2** (Figure 1) as Leu^10^-Val^11^ replacement (LVRs) **3** (Figure 1), which act as rennin [7], and D-Ala-D-Ala ligase inhibitors [8]. The γ-*N*-acylamino-β-hydroxyphosphonic acids **4** (Figure 1) have been used as autotoxin (ATX) inhibitors [9]. Additionally, the γ-amino-β-hydroxyphosphonic acids have been also used as potent sphingosine-1-phosphate (S1P) receptors [10], and as polysaccharide fragments [11,12].

In view of the different biological and chemical applications of the γ-amino-β-hydroxyphosphonate phosphonic acid derivatives, in the last years the development of suitable synthetic methodologies for their preparation in diastereoisomerically pure form has been a topic of great interest in several research groups [13,14,15,16,17,18,19,20,21,22,23,24,25,26,27,28,29]. In this context, several protocols for efficient diastereoselective synthesis of γ-amino-β-hydroxyphosphonic acids and derivatives have emerged, including ring opening of epoxides [30,31,32,33], type aldol reactions of α-aminoaldehydes with dialkyl methylphosphonates [7,8,34,35,36,37,38,39,40,41], catalytic asymmetric aminohydroxylation of β,γ-unsaturated phosphonates [42,43,44], and diastereoselective reduction of γ-amino-β-ketophosphonates [45,46,47].

Recently, we reported the synthesis of phosphostatine and phosphoepistatine [48,49] *via* a high diastereoselective reduction of γ-amino-β-ketophosphonates readily obtained from L-amino acids [50,51,52]. In order to establish a general methodology for the synthesis of *syn*-γ-amino-β-hydroxyphosphonates derived from L-amino acids, in this paper we would like to report the synthesis of γ-amino-β-ketophosphonates **6** and **10** derived from L-proline and L-serine, respectively, and their highly diastereoselective reduction.

## 2. Results and Discussion

**Scheme 1:**
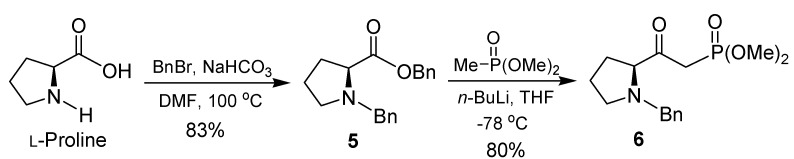
Preparation of β-ketophosphonate **6**.

In our initial study, the synthesis of (*S*)-*N*-benzyl-*O*-benzylpyrrolidine-2-carboxylate (**5**) was carried out by treatment of L-proline with benzyl bromide and K_2_CO_3_ in refluxing ethanol [50], however under this conditions a disappointing poor yield was obtained. For that reason, we decided to examine the methodology developed by Overman and co-workers [53] as a potentially more efficient and practical route to compound **5**. Thus, treatment of L-proline with benzyl bromide and NaHCO_3_ in *N*,*N*-dimethylformamide (DMF) at 100 °C provided the corresponding *N*-benzyl *O*-benzyl proline **5** in 83% yield. Nevertheless, with the *O*-benzyl ester **5** in our hands, we focused our attention on the transformation to β-ketophosphonate **6.** Thus, reaction of **5** with three equivalents of the lithium salt of dimethyl methylphosphonate at -78 °C in THF afforded the corresponding *N*-benzylamino-β-ketophosphonate **6** in 80% yield (Scheme 1).

**Scheme 2:**
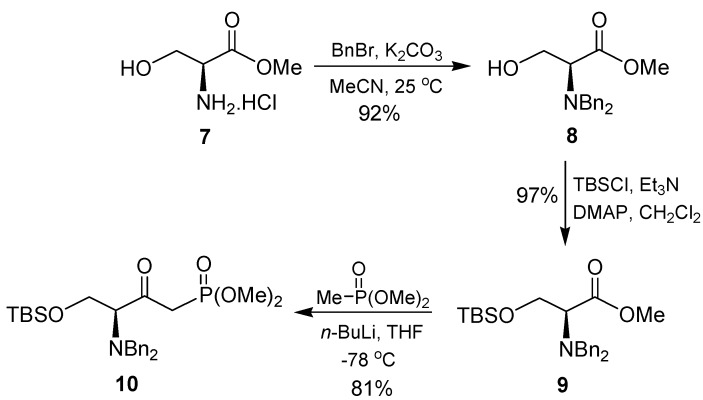
Preparation of β-ketophosphonate **10**.

On the other hand, the reaction of hydrochloride salt of methyl ester of L-serine **7** readily obtained from commercial source or by treatment of L-serine with thionyl chloride in refluxing methanol, with benzyl bromide in the presence of K_2_CO_3_ in acetonitrile at room temperature gave the *N*,*N*-dibenzyl ester **8** in 92% yield. Subsequent treatment with *tert*-butyldimethylsilyl chloride (TBSCl) in the presence of triethylamine and catalytic amounts of 4-*N*,*N*-dimethylaminopyridine (DMAP) in dichloromethane produced the full protected L-serine **9** in 97% yield [54]. *O*-protection in **8** with TBSCl and imidazole in DMF proceed in poor yield. Finally, reaction of **9** with the lithium salt of dimethyl methylphosphonate at -78 °C in THF provided the corresponding γ-*N,N*-dibenzylamino-β-ketophosphonate **10** in 81% yield (Scheme 2).

Having efficiently prepared the β-ketophosphonates **6** and **10**, we turned our attention to the diastereoselective reduction of the carbonyl groups to obtain the corresponding γ*-N*-dibenzylamino- and γ*-N,N*-dibenzylamino-β-hydroxyphosphonates *syn*-**11** and *syn*-**13**, respectively. For this propose we choose NaBH_4_, LiBH_4_, DIBAL-H and catecholborane (CB) as the reducing agents, according to our previous results. Diastereoisomeric excess of the reduction of the β-ketophosphonates **6** and **10** were determined by means of ^31^P-NMR. In fact, the signals for the diastereoisomers *syn* were more shielded than for the diastereoisomers *anti*. Conditions, yields and diastereoisomeric ratio are summarized in Table 1 and Table 2.

**Table 1:** Diastereoselective reduction of β-ketophosphonate **6**.

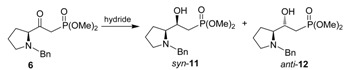
**Entry**	**Hydride**	**Conditions **		**Yield (%)^a^**	***syn-* 11: *anti-* 12 ^b^**
1	NaBH_4_		MeOH, 25 °C	70	69:31
2	LiBH_4_		THF, -78 °C	69	75:25
3	DIBAL-H		THF, -78 °C	69	79:21
4	CB		THF, -78 °C	78	≥96:4

^a^ Determined after purification*;* ^b^ *syn*:*anti* ratios have been determined on the crude products using ^31^P-NMR.

As shown in the Table 1, when the reduction of β-ketophosphonate **6** was carried out with NaBH_4_ in methanol (entry 1, Table 1), a mixture of the γ-amino-β-hydroxyphosphonates *syn*-**11** and *anti*-**12** in a 69:31 ratio in favor of *syn*-**11** was obtained in good yield. The reduction of β-ketophosphonate **6** with LiBH_4_ and DIBAL-H afforded the mixture of the diastereoisomers *syn*-**11** and *anti*-**12** in 69% yield and ratios of 75:25 and 79:21, respectively (entries 2 and 3, Table 1). Finally, the reduction of β-ketophosphonate **6** with catecholborane (CB) in THF at -78 °C (entry 4, Table 1), provided the corresponding γ-amino-β-hydroxyphosphonates in 78% yield, with the *syn:anti* ratio ≥96:4 (the diastereoisomer *anti*-**12** was not observed by ^31^P-NMR).

**Table 2:** Diastereoselective reduction of β-ketophosphonate **10**.

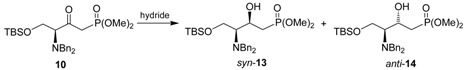
**Entry**	**Hydride**	**Conditions**	**Yield (%)^a^**	***syn-*13: *anti-*14^b^**
1	NaBH_4_	MeOH, 25 °C	70	81:19
2	LiBH_4_	THF, -78 °C	75	82:18
3	DIBAL-H	THF, -78 °C	91	88:12
4	CB	THF, -78 °C	87	≥96:4

^a^ Determined after purification*;* ^b^ *syn:anti* ratios have been determined on the crude products using ^31^P-NMR.

Under similar conditions, the reduction of γ-*N,N*-benzylamino-β-ketophosphonate **10** with NaBH_4_ and LiBH_4_ as reducing agents provided the mixture of the γ-*N,N*-benzylamino-β-hydroxyphosphonates *syn*-**13** and *anti*-**14** in good yield and ratios of 81:19 and 82:18, respectively, (entries 1 and 2, Table 2). A better diastereoselectivity was observed when the β-ketophosphonate **10** was reduced with DIBAL-H in THF at -78 °C (entry 3, Table 2). Finally, reduction of **10** with catecholborane in THF at -78 °C (entry 4, Table 2), afforded the γ-*N,N*-dibenzylamino-β-hydroxyphosphonates in 87% yield, with a *syn: anti* ratio ≥96:4 (the diastereoisomer *anti*-**14** was not observed by ^31^P-NMR). The absolute configuration of the new stereogenic center in *syn*-**11**, *anti*-**12**, *syn*-**13** and *anti*-**14** was assigned by analogy with other γ-amino-β-hydroxyphosphonates obtained in our laboratory.

The formation of the γ-amino-β-hydroxyphosphonates *syn*-**11** and *syn*-**13** as major diastereoisomer in the reduction of the β-ketophosphonates **6** and **10**, respectively, with catecholborane, we propose that the reduction might took place under non-chelation or Felkin-Anh model control [55,56,57,58], and that the bulkiness of the *N*-benzylamino- and *N,N*-dibenzylamino- groups in the β-ketophosphonates **6** and **10**, are sufficient to simultaneously limit the rotamer populations around the hinge bounds adjacent to the carbonyl group blocking the *re* face of carbonyl group and, thereby allowing the addition of hydride to take in a diastereoselective manner by the *si* face (Figure 1).

**Figure 2:**
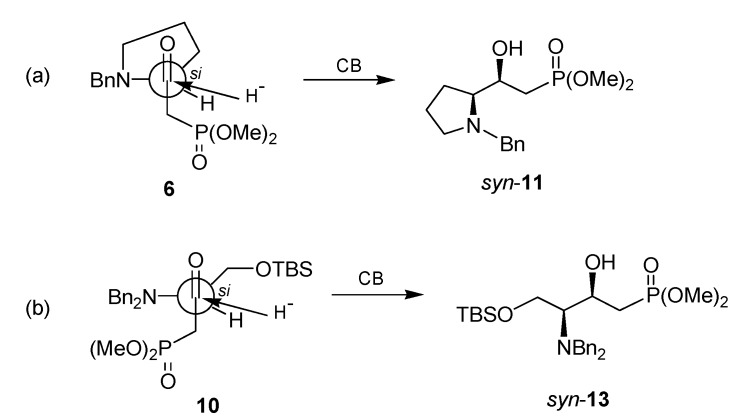
Reduction of the β-ketophosphonates **6** and **10** by non-chelation control.

## 3. Experimental

### 3.1. General Procedures

Optical rotations were taken on a Perkin-Elmer 241 polarimeter in a 1 dm tube; concentrations are given in g/100 mL. For flash chromatography, silica gel 60 (230–400 mesh ASTM, Merck) are used. ^1^H-NMR spectra were recorded on a Varian INOVA 400 (at 400 MHz), ^13^C- (100 MHz) and ^31^P-NMR on a Varian Mercury 200 instrument. HRMS spectra were recorded on a JEOL JMS-700 instrument. Flasks, stirring bars, and hypodermic needles used for the generation of organometallic compounds were dried for ca. 12 h at 120 °C and allowed to cool in a desiccator over anhydrous calcium sulfate. Anhydrous solvents (ethers) were obtained by distillation from benzophenone ketyl. The preparation and spectroscopic data for the compounds (*S*)-*N*-benzyl-*O*-benzylpyrrolidine-2-carboxylate (**5**) [53], (*S*)-methyl-2-(dibenzylamino)-3-hydroxypropanoate(**8**) [59] and (*S*)-methyl-3-(*tert*-butyldimethyl-silyloxy)-2-(dibenzylamino) propanoate (**9**) [59], have all been described in the cited literature.

*(S)-Dimethyl-2-(1-benzylpyrrolidin-2-yl)-2-oxoethylphosphonate* (**6**). A solution of dimethyl methylphosphonate (830 mg, 6.8 mmol) in anhydrous THF (50 mL), was cooled at -78 °C before the slow addition of *n*-BuLi 2.35 M in hexanes (2.9 mL, 6.9 mmol). The resulting solution was stirred at -50 °C for 1.5 h and then cooled at -78 °C, followed by the addition of a solution of benzyl ester **5** (500 mg, 1.7 mmol) in anhydrous THF (50 mL). The reaction mixture was stirred at -78 °C for 4 h before the addition of a saturated solution of NH_4_Cl. The solvent was evaporated under reduced pressure, the residue was dissolved in water (30 mL) and extracted with ethyl acetate (3 × 30 mL). The combined organic extracts were dried over anhydrous Na_2_SO_4_, filtered and evaporated under reduced pressure. The crude product was purified by column chromatography using hexane-ethyl acetate (50:50) as eluent to afford the desired product (420 mg, 80% yield) as a viscous oil. [α]_D_ = **-**1.3 (c = 1.37, CHCl_3_). ^1^H-NMR (CDCl_3_) δ 1.77–2.15 (m, 4H), 2.36 (m, 1H), 2.98 (dd, *J* = 21.2, 15.0 Hz, 1H, CH_2_P), 3.07 (m, 1H), 3.27 (dd, *J* = 9.2, 6.6 Hz, 1H, CHN), 3.42 (dd, *J* = 21.2, 15.0, Hz, 1H, CH_2_P), 3.48 (system AB, *J* = 15.0 Hz, 1H, CH_2_Ph), 3.75 (d, *J* = 11.2 Hz, 3H, (CH_3_O)_2_P), 3.77 (d, *J* = 11.2 Hz, 3H, (CH_3_O)_2_P), 3.82 (system AB, *J* = 15.0 Hz, 1H, CH_2_Ph), 7.23–7.36 (m, 5 H, H_arom_); ^13^C-NMR (CDCl_3_) 23.9 (*C*H_2_CH_2_), 28.5 (*C*H_2_CH), 35.7 (d, *J* = 133.6 Hz, *C*H_2_P), 53.1 [(*C*H_3_O)_2_P], 53.3 [(*C*H_3_O)_2_P], 54.2 (*C*H_2_N), 59.5 (*C*H_2_Ph), 73.7 (*C*HN), 127.4 (*C_para_*), 128.4 (*C_meta_*), 129.2 (*C_ortho_*), 138.4 (*C_ipso_*), 204.8 (*C*=O); ^31^P-NMR (CDCl_3_) δ 25.94; HRMS (CI, CH_4_) calculated for C_15_H_23_O_4_NP (MH^+^) 312.1365, found 312.1287.

*(S)-Dimethyl-4-(tert-butyldimethylsilyloxy)-3-N,N-(dibenzylamino)-2-oxobutylphosphonate* (**10**)*.* A solution of dimethyl methylphosphonate (3.30 g, 26.6 mmol) in anhydrous THF (125 mL), was cooled at -78 °C before the slowly addition of *n*-BuLi 2.15 M in hexanes (12.7 mL, 27.3 mmol). The resulting solution was stirred at -50 °C for 1.5 h and then cooled at -78 °C followed by the addition of a solution of benzyl ester **9** (2.75 g, 6.7 mmol) in anhydrous THF (125 mL). The reaction mixture was stirred at -78 °C for 4 h before the addition of a saturated solution of NH_4_Cl. The solvent was evaporated under reduced pressure, the residue was dissolved in water (30 mL) and extracted with ethyl acetate (3 × 30 mL). The combined organic extracts were dried over anhydrous Na_2_SO_4_, filtered and evaporated under reduced pressure. The crude product was purified by column chromatography using hexane-ethyl acetate (50:50) as eluent to give the desired product (2.7 g, 81% yield) as a viscous oil. [α]_D_ = **-**56.0 (c = 1.17, CHCl_3_). ^1^H-NMR (CDCl_3_) δ 0.09 (s, 3H, (CH_3_)_2_Si), 0.12 (s, 3H, (CH_3_)_2_Si), 0.93 (s, 9H, (CH_3_)_3_C), 2.99 (dd, *J* = 21.9 Hz, *J* = 14.5 Hz, 1H, CH_2_P), 3.48 (dd, *J* = 21.9 Hz, *J* = 14.5 Hz, 1H, CH_2_P), 3.56 (d, *J* = 11.2 Hz, 3H, (CH_3_O)_2_P), 3.63 (d, *J* = 11.2 Hz, 3H, (CH_3_O)_2_P), 3.65 (t, *J* = 6.0 Hz, 1H, CHN), 3.78 (system AB, *J* = 13.4 Hz, 2H, CH_2_Ph), 3.84 (system AB, *J* = 13.4 Hz, 2H, CH_2_Ph), 4.03 (dd, *J* = 11.0 Hz, *J* = 6.1 Hz, 1H, CH_2_OSi), 4.13 (dd, *J* = 11.0 Hz, *J* = 6.1 Hz, 1H, CH_2_OSi), 7.23–7.35 (m, 10 H, H_arom_); ^13^C-NMR (CDCl_3_) δ -5.3 [(*C*H_3_)_2_Si], -5.2 [(*C*H_3_)_2_Si], 18.4 [*C*(CH_3_)_3_], 26.2 [*C*H_3_)_3_C], 38.6 (d, *J* = 130.6 Hz, *C*H_2_P), 52.9 [d, *J* = 6.1 Hz, (*C*H_3_O)_2_P)], 52.9 [d, *J* = 6.0 Hz, (*C*H_3_O)_2_P], 55.4 (*C*H_2_Ph), 60.1 (*C*H_2_OSi), 67.44 (*C*HN), 127.4 (*C_para_*), 128.5 (*C_meta_*), 129.2 (*C_ortho_*), 139.4 (*C_ipso_*), 201.8 (d, *J* = 6.1 Hz, *C*=O); ^31^P-NMR (CDCl_3_) δ 24.30; HRMS (CI, CH_4_) calculated for, C_26_H_41_O_5_NPSi (MH^+^) 506.2492, found 506.2575

*General procedure for the reduction of* *β-ketophosphonates*(*S*)**-6** *and* (*S*)**-10** *with NaBH_4_*. To a solution of β-ketophosphonate (*S*)-**6** or (*S*)-**10** (1.0 eq.) in methanol (40 mL) at 0 °C was added NaBH_4_ (4.0 equiv.). After 5.0 h, the solvent was evaporated and the residue was diluted with H_2_O and extracted with ethyl acetate (3 × 30 mL). The organic layer was dried over Na_2_SO_4_ and evaporated in vacuum. The crude was analyzed by ^1^H- and ^31^P-NMR and purified by column chromatography.

*General procedure for the reduction of* *β-ketophosphonates*(*S*)-**6** and(*S*)**-10** with LiBH_4_, DIBAL-H and catecholborane (CB)**.** To a solution of β-ketophosphonate (*S*)-**6** or (*S*)-**10** (1.0 eq.) in anhydrous THF (50 mL) was added (2.0 equiv.) of reducing agent at -78 °C. The reaction mixture was stirred for 5.0 h at -78 °C, and then was quenched with saturated solution of NH_4_Cl and extracted with ethyl acetate (3 × 40 mL). The organic layer was dried over Na_2_SO_4_ and evaporated in vacuum. The crude was analyzed by ^1^H- and ^31^P-NMR and purified by column chromatography.

*(2S)-1-Benzylpyrrolidin-2-yl)-(2R)-hydroxyethylphosphonate* (*syn-***11**). Following the general procedure, β-ketophosphonate **6** (100 mg, 0.32 mmol) in anhydrous THF (20 mL), was treated with catecholborane (CB), 1 M in THF, (1.5 mL, 1.5 mmol). After work up and chromatographic purification gave (78 mg, 78% yield) of β-hydroxyphosphonate *syn-***11** as a viscous oil. [α]_D_ = -2.0 (c = 1.02, CHCl_3_); ^1^H-NMR (CDCl_3_) δ 1.24-1.90 (m, 7H), 2.48-2.58 (m, 1H,CH_2_P), 2.90-3.10 (m, 1H, CH_2_P), 3.421-3.614 (m,1H), 3.48 (system AB, *J* = 13.0 Hz, 1H, CH_2_Ph), 3.68 (d, *J* = 11.0 Hz, 3H, (CH_3_O)_2_P), 3.76 (d, *J* = 11.2 Hz, 3H, (CH_3_O)_2_P), 4.02 (system AB, *J* = 13.0 Hz, 1H, CH_2_Ph), 7.23–7.36 (m, 10 H, H_arom_); ^13^C-NMR (CDCl_3_) 21.6 (*C*H_2_CH_2_), 29.5 (*C*H_2_CH), 330.1 (d, *J* = 133.6 Hz, *C*H_2_P), 36.64 (*C*H_2_N), 46.8 (N*C*H_2_Ph), 51.7 [(*C*H_3_O)_2_P], 53.2 [(*C*H_3_O)_2_P], 68.20 (*C*HN), 68.3 (*C*HOH), 127.4 (*C_para_*), 128.4 (*C_meta_*), 129.2 (*C_ortho_*), 138.4 (*C_ipso_*); ^31^P-NMR (CDCl_3_) δ 35.3; HRMS (CI, CH_4_) calculated for C_15_H_25_O_4_NP (MH^+^) 314.1521, found 314.1506.

*Dimethyl-(2R,3S)-4-(tert-butyldimethylsilyloxy)-3-N,N-(dibenzylamino)-2-hydroxybutyl-phosphonate*

(*syn-***13**). Following the general procedure, (180 mg, 0.37mmol) of β-ketophosphonate **10** in anhydrous THF (20 mL), was treated with catecholborane (CB) 1 M in THF (1.5 mL, 1.5 mmol) of. After work up and chromatographic purification, (150 mg, 87% yield) of β-hydroxyphosphonate *syn-***13** was obtained as a viscosus oil. [α]_D_ = **+**17.1 (c = 1.01, CHCl_3_); ^1^H-NMR (CDCl_3_) δ 0.12 (s, 3H, (CH_3_)_2_Si), 0.12 (s, 3H, (CH_3_)_2_Si), 0.93 (s, 9H, (CH_3_)_3_C), 1.79 (ddd, *J* = 20.0, 15.1, 5.8 Hz, 1H, CH_2_P), 1.95 (ddd, *J* = 20.0 Hz, 15.1, 5.8 Hz, 1H, CH_2_P), 2.64 (m 1H), 3.57 (system AB, *J* = 13.4 Hz, 2H, CH_2_Ph), 3.67 (d, *J* = 11.0 Hz, 3H, (CH_3_O)_2_P), 3.72 (d, *J* = 11.0 Hz, 3H, (CH_3_O)_2_P), 3.90 (m, 2H, CH_2_OSi), 4.00 (system AB, *J* = 13.4 Hz, 2H, CH_2_Ph), 4.03–4.13 (m, 1H), 7.22–7.33 (m, 10 H, H_arom_); ^13^C-NMR (CDCl_3_) δ -5.4 ((*C*H_3_)_2_Si), -5.3 ((*C*H_3_)_2_Si), 18.3 (*C*(CH_3_)_3_), 26.1 (*C*H_3_)_3_C), 30.5 (d, *J* = 141.2 Hz, *C*H_2_P), 52.5 (d, *J* = 13.6 Hz, 2C, (*C*H_3_O)_2_P), 55.9 (*C*H_2_Ph), 59.6 (*C*H_2_OSi), 63.9 (*C*HOH), 64.1 (CHN), 127.4 (*C_para_*), 128.6 (*C_meta_*), 129.4 (*C_ortho_*); 139.4 (C*_ipso_*); ^31^P-NMR (CDCl_3_) δ 33.92. HRMS (CI, CH_4_) calculated for C_26_H_43_O_5_NPSi (MH^+^) 508.2648, found 508.2672.

## 4. Conclusions

In conclusion, we have found that the reduction of *N,N*-disubstituted-γ-amino-β-ketophosphonates readily obtained from the appropriate L-amino acids, with catecholborane (CB) afforded the *syn*-γ-amino-β-hydroxyphosphonates as principal diastereoisomers, which could be used as template compounds for the synthesis of molecules with biological and chemical interest.
